# ﻿Lignicolous freshwater ascomycetes from Thailand: Introducing *Dematipyriformamuriformis* sp. nov., one new combination and two new records in Pleurotheciaceae

**DOI:** 10.3897/mycokeys.93.87797

**Published:** 2022-09-29

**Authors:** Dan-Feng Bao, Darbhe J. Bhat, Saranyaphat Boonmee, Kevin D. Hyde, Zong-Long Luo, Sarunya Nalumpang

**Affiliations:** 1 College of Agronomy and Biosciences, Dali University, Dali 671003, Yunnan, China; 2 Center of Excellence in Fungal Research, Mae Fah Luang University, Chiang Rai 57100, Thailand; 3 Department of Entomology & Plant Pathology, Faculty of Agriculture, Chiang Mai University, Chiang Mai 50200, Thailand; 4 128/1-J, Azad Housing Society, Curca, Goa Velha-403108, India; 5 School of Science, Mae Fah Luang University, Chiang Rai 57100, Thailand

**Keywords:** 1 new combination, 1 new taxon, freshwater fungi, phylogeny, Pleurotheciales, taxonomy

## Abstract

During the study of lignicolous freshwater fungi from Thailand, three pleurotheciaceous species were collected from freshwater habitats in Thailand. Two were identified as *Pleurotheciumaquaticum* and *Rhexoacrodictysfimicola*, and the third is a new species *Dematipyriformamuriformis* sp. nov.. *Rhexoacrodictys* is accepted in Pleurotheciaceae based on phylogenetic analysis. *Rhexoacrodictysnigrospora* is transferred to *Dematipyriforma* based on phylogenetic analysis and morphological characters. *Pleurotheciumaquaticum* and *Rhexoacrodictysfimicola* are reported from Thailand for the first time.

## ﻿Introduction

Pleurotheciales was introduced by Réblová et al. (2016) to accommodate a single family Pleurotheciaceae. The order was originally placed in Hypocreomycetidae by Réblová et al. (2016). Hongsanan et al. (2017) showed that Pleurotheciales clustered with Conioscyphales, Fuscosporellales and Savoryellales in a monophyletic clade within Sordariomycetes. Hence, they transferred Pleurotheciales to a newly introduced subclass Savoryellomycetidae based on phylogenetic analysis and the placement has been confirmed and accepted by Dayarathne et al. (2019) and Hyde et al. (2020a).

Pleurotheciaceae was introduced by Réblová et al. (2016) with *Pleurothecium* Höhn. as the type genus. Currently, *Adelosphaeria*, *Anapleurothecium*, *Coleodictyospora*, *Dematipyriforma*, *Helicoascotaiwania*, *Melanotrigonum*, *Neomonodictys*, *Phaeoisaria*, *Phragmocephala*, *Pleurotheciella*, *Pleurothecium*, *Saprodesmium*, and *Sterigmatobotrys* are accepted in this family (Hyde et al. 2020a; Wijayawardene et al. 2020; Dong et al. 2021). The sexual morphs of Pleurotheciaceae share dark, papillate, perithecial, astromatic, immersed to superficial ascomata, unitunicate asci with a distinct non-amyloid apical annulus, and fusiform to ellipsoidal, septate, hyaline ascospores (Réblová et al. 2016; Luo et al. 2018a; Hyde et al. 2020a). The asexual morphs of Pleurotheciaceae are diverse in morphology, comprising *acrodictys*-like (*Monotosporella*), (Hyde and Yanna 2002; Sadowski et al. 2012), *helicoön*-like (*Helicoascotaiwania*, Dayarathne et al. 2019; Réblová et al. 2020), *monodictys*-like (*Neomonodictys*, Hyde et al. 2020b) and *dactylaria*-like taxa (*Pleurotheciella*, *Phaeoisaria* and *Pleurothecium*, Réblová et al. 2016; Luo et al. 2018a). Species in Pleurotheciaceae are cosmopolitan with a worldwide distribution and have been reported from both aquatic and terrestrial habitats (Réblová et al. 2016, 2020; Hernandez-Restrepo et al. 2017; Luo et al. 2018a, 2019; Hyde et al. 2020a, b).

In this study, three new collections are placed in *Dematipyriforma*, *Rhexoacrodictys* and *Pleurothecium* respectively. The monotypic genus *Dematipyriforma* was introduced to accommodate an endophytic species, *D.aquilaria* which was collected from wood of *Aquilariacrassna* (Sun et al. 2017). *Dematipyriforma* was originally placed in Savoryellales (Sun et al. 2017). However, Dong et al. (2021) showed that *Dematipyriforma* clustered within Pleurotheciales and sister to *Rhexoacrodictys* and *Saprodesmium*. In addition, the morphology of *Dematipyriforma* is similar to *Neomonodictys* in Pleurotheciales. Therefore, they transferred *Dematipyriforma* to Pleurotheciales based on phylogenetic analysis and morphological characteristics. *Rhexoacrodictys* was introduced by Baker et al. (2002) to accommodate species previously identified as *Acorcdictys* (i.e., *A.erecta*, *A.fimicola*, *A.fuliginosa* and *A.queenslandica*) and wherein *Rhexoacrodictyserecta* was designated as the type. Two additional species *R.martini* and *R.broussonetiae* were subsequently added to the genus based on morphological characteristics (Delgado 2009; Xiao et al. 2018). While *R.martini* and *R.queenslandica* were transferred to *Distoseptispora* and *Junewangia* based on phylogenetic analysis (Xia et al. 2017). Currently, four species are accepted in *Rhexoacrodictys*. *Pleurothecium* was established by Höhnel (1919) with *P.recurvatum* (Morgan) Höhn as type species. *Pleurothecium* species are characterized by macronematous, mononematous, septate, brown conidiophores, polyblastic, sympodially extended, denticulate conidiogenous cells and solitary, septate, hyaline or pigmented or bicolored conidia (Goos 1969; Matsushima 1975, 1980; Subramanian and Bhat 1989; Matsushima and Matsushima 1996; Cooper 2005; Arzanlou et al. 2007; Wu and Zhang 2009; Réblová et al. 2012; Monteiro et al. 2016; Luo et al. 2018a). Presently, 11 species are accepted in the genus. Most *Pleurothecium* species are reported as saprobes from freshwater or terrestrial habitats (Wu and Zhang 2009; Réblová et al. 2012; Monteiro et al. 2016; Luo et al. 2018a).

We are currently investigating the diversity of lignicolous freshwater fungi from the Greater Mekong Subregion (Hyde et al. 2016). Thailand is an area of the Greater Mekong Subregion with rich fungal biodiversity. Freshwater fungi have been studied in Thailand over several decades initiated by Tubaki et al. (1983) who found 40 Ingoldian fungi in the stream foams. Many new freshwater taxa have since been reported in Thailand, especially a large number of lignicolous freshwater ascomycetes (Sivichai et al. 1998, 2000, 2002; Jones et al. 1999; Sivichai and Boonyene 2004; Zhang et al. 2011; Luo et al. 2019; Dong et al. 2020; Calabon et al. 2021, 2022). Until 2020, more than 302 freshwater taxa had been reported from Thailand (Zhang et al. 2011; Calabon et al. 2021). In this study, we introduce three taxa of Pleurotheciaceae, collected from freshwater habitats in Thailand. With phylogenetic analysis of ITS, LSU, SSU, RPB2 and TEF1-α sequence data, they are placed in *Dematipyriforma*, *Pleurothecium* and *Rhexoacrodictys* within Pleurotheciaceae. Of these three species, one is identified as *Pleurotheciumaquaticum*, one as *Rhexoacrodictysfimicola*, and the third as a new species in *Dematipyriforma*. In addition, *Rhexoacrodictysnigrospora* is transferred to *Dematipyriforma* based on morphological and phylogenetic evidence.

## ﻿Materials and methods

### ﻿Collection, isolation and morphology

Submerged decaying woods were collected from the streams in Thailand. The sample incubation, examination and morphological studies were referred to the methods described by Luo et al. (2018b). Single spore isolations were followed the methods outlined by Senanayake et al. (2020). Specimens (dry wood with fungal material) were deposited in the herbarium of Mae Fah Luang University (**MFLU**), Chiang Rai, Thailand and Herbarium of Cryptogams Kunming Institute of Botany Academia Sinica (**KUN-HKAS**). Pure cultures were deposited in Mae Fah Luang University Culture Collection (**MFLUCC**) and Kunming Institute of Botany culture collection (**KUNCC**). Faces of Fungi and Index Fungorum numbers were registered as outlined in Jayasiri et al. (2015) and Index Fungorum (2022). The descriptions are added to it GMS database (Chaiwan et al. 2021).

### ﻿DNA extraction, PCR amplification and sequencing

Genomic DNA was extracted from fungal mycelium (*Rhexoacrodictyserecta* and *Pleurotheciumaquaticum*) or directly from the conidiamatal tissue thalli of fungi (*Dematipyriformamuriformis*) as outlined by Wanasinghe et al. (2018). The Ezup Column Fungi Genomic DNA Purification Kit (Sangon Biotech, China) was used to extract DNA following the manufacturer’s instructions. ITS, LSU, SSU, RPB2 and TEF1-α gene regions were amplified using the primer pairs ITS5/ITS4, LR0R/LR7, NS1/NS4, fRPB2-5F/fRPB2-7cR and 983F/2218R, respectively (Vilgalys and Hester 1990; White et al. 1990; Liu et al. 1999). The amplification was performed in a 25 μl reaction volume containing 9.5 μl ddH_2_O, 12.5 μl 2 × Taq PCR Master Mix with blue dye (Sangon Biotech, China), 1 μl of DNA template and 1 μl of each primer (10 μM). The amplification condition for ITS, LSU, SSU, RPB2 and TEF1-α were followed Luo et al. (2018b). DNA sequencing of PCR products were carried out using the above-mentioned PCR primers at Tsingke Biological Engineering Technology and Services Co. (Yunnan, P.R. China).

### ﻿Phylogenetic analyses

The taxa used in the phylogenetic analysis were obtained from previous studies (Table 1) (Hernandez-Restrepo et al. 2017; Luo et al. 2018a, 2019; Dayarathne et al. 2019; Hyde et al. 2020b; Réblová et al. 2020; Boonmee et al. 2021; Dong et al. 2021) and downloaded from GenBank. SEQMAN v. 7.0.0 (DNASTAR, Madison, WI) was used to assemble the consensus sequences and MAFFT v.7 online program (http://mafft.cbrc.jp/alignment/server/) was used to align the sequences (Katoh et al. 2019). BioEdit was used to manually adjust the alignments and the alignment fasta file was converted to Phylip format by Alivew (Hall 2021; Larsson 2014).

**Table 1.** Taxa used in this study; the ex-type strains were indicated in bold, newly generated sequences are indicated by * after the species name.

Species	Strain number	GenBank accession number				
		ITS	LSU	SSU	RPB2	TEF1-α
***Adelosphaeriacatenata***	**CBS 138679**	**KT278721**	**KT278707**	**KT278692**	**KT278743**	–
***Anapleurotheciumbotulisporum***	**CBS 132713**	**KY853423**	**KY853483**	–	–	–
*Ascotaiwanialignicola*	NIL00005	HQ446341	HQ446364	HQ446284	HQ446419	HQ446307
*Ascotaiwaniasawadae*	SS00051	HQ446340	HQ446363	HQ446283	HQ446418	HQ446306
***Bactrodesmiastrumobovatum***	**FMR 6482**	**FR870264**	**FR870266**	–	–	–
***Bactrodesmiastrumpyriforme***	**FMR 10747**	**FR870263**	**FR870265**	–	–	–
*Bactrodesmiumabruptum*	CBS 144404	MN699391	MN699408	MN699365	MN704288	MN704313
*Bactrodesmiumleptopus*	CBS 144542	MN699388	MN699423	MN699374	MN704297	MN704321
*Bactrodesmiumobovatum*	CBS 144077	MN699395	MN699424	MN699375	MN704298	MN704322
*Canalisporiumexiguum*	SS00809	GQ390296	GQ390281	GQ390266	HQ446436	–
***Canalisporiumgrenadoideum***	**SS03615**	–	**GQ390267**	**GQ390252**	**HQ446420**	**HQ446309**
*Coleodictyosporamuriformis*	MFLUCC 18–1243	MW981642	MW981648	MW981704	–	–
*Coleodictyosporamuriformis*	MFLUCC 18–1279	MW981643	MW981649	MW981705	–	–
*Conioscyphahoehnelii*	FMR 11592	KY853437	KY853497	HF937348	–	–
***Conioscyphalignicola***	**CBS 335.93**	–	**AY484513**	**JQ437439**	**JQ429260**	–
***Conioscyphaperuviana***	**ILL41202**	–	**KF781539**	–	–	–
***Conioscyphapleiomorpha***	**FMR 13134**	**KY853438**	**KY853498**	–	–	–
***Dematipyriformaaquilaria***	**CGMCC 3.17268**	**KJ138621**	**KJ138623**	**KJ138622**	–	–
Dematipyriformamuriformis ^*^	**MFLU 21–0146**	**OM654773**	**OM654770**	–	–	**OM672032**
***Dematipyriformanigrospora***	**MFLUCC 21-0096**	**MZ538524**	**MZ538558**	–	–	**MZ567100**
*Dematipyriformanigrospora*	MFLUCC 21-0097	MZ538525	MZ538559	MZ538574	MZ567113	MZ567101
***Fuscosporellapyriformis***	**MFLUCC 16–0570**	–	**KX550896**	**KX550900**	**KX576872**	–
***Helicoascotaiwaniafarinosa***	**ILLS 53605**	–	**AY094189**	–	–	–
*Helicoascotaiwaniafarinosa*	DAOMC 241947	JQ429145	JQ429230	–	–	–
***Helicoascotaiwanialacustris***	**CBS 145963**	–	**MN699430**	**MN699382**	**MN704304**	**MN704329**
*Helicoascotaiwanialacustris*	CBS 145964	MN699400	MN699431	MN699383	MN704305	–
*Helicoascotaiwanialacustris*	CBS 146144	MN699401	MN699432	MN699384	MN704306	–
*Leotialubrica*	AFTOL-ID1	DQ491484	AY544644	AY544746	DQ470876	DQ028596
*Melanotrigonumovale*	CBS 138815	KT278722	KT278711	KT278698	KT278747	–
*Microglossumrufum*	AFTOL-ID 1292	–	DQ470981	DQ471033	DQ470933	DQ471104
*Monotosporellasetosa*	HKUCC3713	–	AF132334	–	–	–
***Mucisporaobscuriseptata***	**MFLUCC 15–0618**	–	**KX550892**	**KX550897**	–	–
***Mucisporaphangngaensis***	**MFLUCC 16–0865**	–	**MG388210**	**MG388207**	–	–
*Neomonodictysmuriformis*	MFLUCC 16–1136	MN644509	MN644485	–	–	MN646856
***Obliquifusoideumguttulatum***	**MFLUCC 18–1233**	MW981645	MW981650	MW981706	–	–
***Parafuscosporellagarethii***	**FF00725.01**	–	**KX958430**	**KX958428**	**KX958432**	–
***Parafuscosporellamoniliformis***	**MFLUCC 15–0626**	–	**KX550895**	**KX550899**	–	–
***Parafuscosporellamucosa***	**MFLUCC 16–0571**	–	**MG388211**	**MG388208**	–	–
***Phaeoisariaaquatica***	**MFLUCC 16–1298**	**MF399237**	**MF399254**	–	**MF401406**	–
*Phaeoisariaclematidis*	MFLUCC 17–1968	MG837022	MG837017	MG837027	–	–
***Phaeoisariafasciculata***	**CBS 127885**	–	**KT278705**	**KT278693**	**KT278741**	–
*Phaeoisariafiliformis*	MFLUCC 18–0214	MK878381	MK835852	MK834785	–	MN200285
***Phaeoisariaguttulata***	**MFLUCC 17–1965**	**MG837021**	**MG837016**	**MG837026**	–	–
***Phaeoisariapseudoclematidis***	**MFLUCC 11–0393**	–	**KP744501**	**KP753962**	–	–
***Phaeoisariasedimenticola***	**CGMCC 3.14949**	–	**JQ031561**	–	–	–
***Phaeoisariasedimenticola***	**S-908**	**MK878380**	**MK835851**	–	–	**MN200284**
*Phaeoisariasparsa*	FMR11939	–	HF677185	–	–	–
*Phragmocephalastemphylioides*	DAOM 673211	KT278730	KT278717	–	–	–
*Pleurotheciellaaquatica*	MFLUCC 17–0464	MF399236	MF399253	MF399220	MF401405	–
***Pleurotheciellacentenaria***	**DAOM 229631**	–	**JQ429234**	**JQ429246**	**JQ429265**	–
*Pleurotheciellafusiformis*	MFLUCC 17–0115	MF399232	MF399249	MF399217	MF401402	–
***Pleurotheciellaguttulata***	**KUMCC 15–0296**	**MF399240**	**MF399257**	**MF399223**	**MF401409**	–
***Pleurotheciellakrabiensis***	**MFLUCC 18–0852**	**MG837018**	**MG837013**	**MG837023**	–	–
***Pleurotheciellalunata***	**MFLUCC 17–0111**	**MF399238**	**MF399255**	**MF399221**	**MF401407**	–
***Pleurotheciellarivularia***	**CBS 125238**	–	**JQ429232**	**JQ429244**	**JQ429263**	–
*Pleurotheciellarivularia*	CBS 125237	–	JQ429233	JQ429245	JQ429264	–
***Pleurotheciellasaprophytica***	**MFLUCC 16–1251**	**MF399241**	**MF399258**	**MF399224**	**MF401410**	–
*Pleurotheciellasubmersa*	MFLUCC 17–1709	MF399243	MF399260	MF399226	MF401412	–
***Pleurotheciellasubmersa***	**MFLUCC 17–0456**	**MF399244**	**MF399261**	**MF399227**	**MF401413**	–
***Pleurotheciellatropica***	**MFLUCC 16–0867**	**MG837020**	**MG837015**	**MG837025**	–	–
***Pleurotheciellauniseptata***	**DAOM 673210**	**KT278729**	**KT278716**	–	–	–
***Pleurotheciumaquaticum***	**MFLUCC 17–1331**	**MF399245**	**MF399263**	–	–	–
*Pleurotheciumaquaticum* ^*^	KUMCC 21-0477	OM654775	OM654772	OM654807	OM672034	OM672033
***Pleurotheciumfloriforme***	**MFLUCC 15–0628**	**NR_156614**	**NG_059791**	–	–	–
*Pleurotheciumobovoideum*	CBS 209.95	EU041784	EU041841	–	–	–
*Pleurotheciumpulneyense*	MFLUCC 16–1293	–	MF399262	MF399228	MF401414	–
*Pleurotheciumrecurvatum*	CBS 138686	–	KT278715	KT278702	–	–
***Pleurotheciumsemifecundum***	**CBS 131271**	–	**JQ429240**	**JQ429254**	**JQ429270**	–
*Rhexoacrodictyserecta*	HSAUPmyr4622	KU999964	KX033556	KX033526	–	–
*Rhexoacrodictyserecta*	IFRD500–016	MT555421	MT559123	MT555735	–	–
*Rhexoacrodictyserecta*	HSAUP myr6489	KU999963	KX033555	KX033525	–	–
*Rhexoacrodictysfimicola*	HMAS 47737	KU999960	KX033553	KX033522	–	–
*Rhexoacrodictysfimicola*	HMAS 42882	KU999962	KX033554	KX033524	–	–
*Rhexoacrodictysfimicola*	HMAS 43690	KU999957	KX033550	KX033519	–	–
*Rhexoacrodictysfimicola* ^*^	MFLUCC 18–0340	OM654774	OM654771	OM654806	–	–
***Saprodesmiumdematiosporium***	**KUMCC 18–0059**	**MW981646**	**MW981647**	**MW981707**	–	–
*Savoryellaaquatica*	SS03801	–	HQ446372	HQ446292	HQ446405	HQ446326
*Savoryellalignicola*	NF00204	–	HQ446378	HQ446300	HQ446413	HQ446334
*Sterigmatobotrysmacrocarpa*	MR2973	–	GU017317	–	–	–
*Sterigmatobotrysrudis*	DAOM 229838	JQ429152	JQ429241	JQ429256	JQ429272	–
***Sterigmatobotrysuniseptata***	**MFLUCC 15–0358**	**MK878379**	**MK835850**	**MK834784**	–	–

Maximum likelihood (ML) analysis generated using the RAxML-HPC2 on XSEDE (v.8.2.8) in the CIPRES Science Gateway (https://www.phylo.org, Stamatakis 2006; Stamatakis et al. 2008; Miller et al. 2010) with rapid bootstrap analysis, followed by 1000 bootstrap replicates, using the GTR+I+G model of evolution.

Bayesian analysis was performed by MrBayes v. 3.2 (Ronquist et al. 2012), best-fit model of DNA evolution for the Bayesian inference analysis was estimated by MrModeltest v. 2.2 (Nylander 2004) and the GTR+I+G model was selected for LSU, ITS, RPB2 and TEF1-α, GTR+G model was selected for SSU. Posterior probabilities (PP) (Rannala and Yang 1996; Zhaxybayeva and Gogarten 2002) was defined by Bayesian Markov Chain Monte Carlo (BMCMC) sampling method in MrBayes v. 3.0b4 (Huelsenbeck and Ronquist 2001). Six simultaneous Markov Chains were run for 50,000,000 generations and trees were sampled every 500^th^ generation (resulting in 100,000 trees). The first 20% trees that represented the burn-in phase were discarded and the remaining 80% (post burn-in) trees used for calculating posterior probabilities (PP) for the majority rule consensus tree.

Phylogenetic trees were visualized with FigTree v. 1.4.2 (Rambaut 2014) and edited in Microsoft Office PowerPoint 2019 (Microsoft Inc., United States). Newly generated sequences in this study were deposited in GenBank.

## ﻿Results

### ﻿Phylogenetic analyses

The dataset of combined ITS, LSU, SSU, RPB2 and TEF1-α sequence data comprises 81 strains with 4257 characters including gaps (ITS: 509 bp, LSU: 1006 bp, SSU: 862 bp, RPB2: 1032 bp, TEF1-α: 848 bp). *Leotialubrica* (AFTOL-ID1) and *Microglossumrufum* (AFTOL-ID 1292) were used as outgroup taxa. RAxML and Bayesian analyses were conducted and resulted in generally congruent topologies. The best RAxML tree with a final likelihood value of –45872.924927 is presented. The matrix had 2433 distinct alignment patterns, with 44.65% undetermined characters or gaps. Estimated base frequencies were as follows: A = 0.234712, C = 0.261626, G = 0.290634, T = 0.213028; substitution rates AC = 1.347806, AG = 2.754719, AT = 1.490447, CG = 1.095887, CT = 6.696475, GT = 1.000000; gamma distribution shape parameter α = 0.316898.

**Figure 1.**
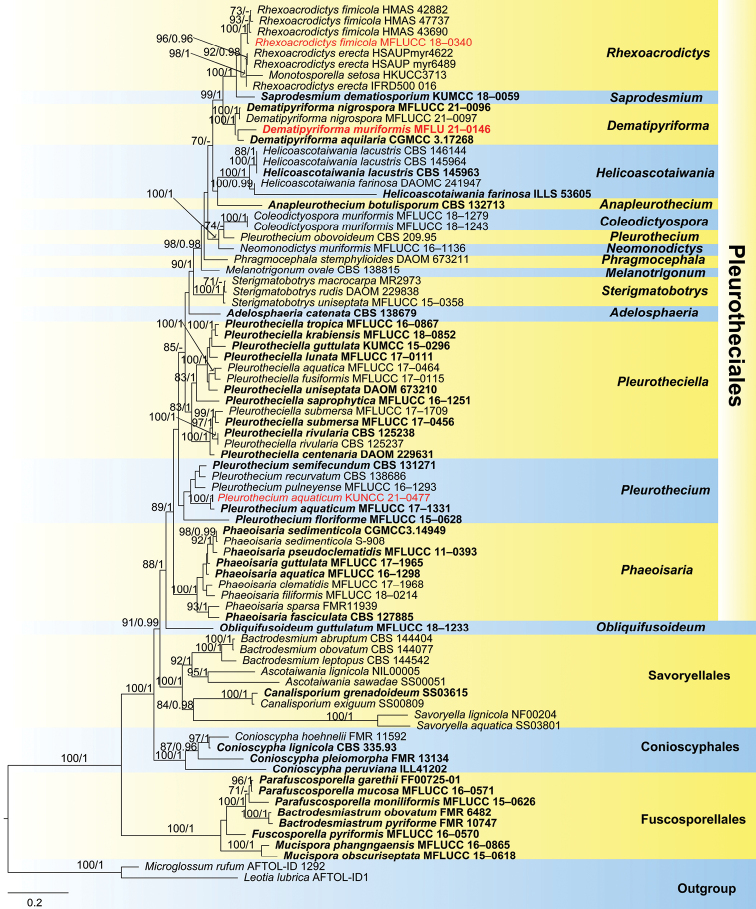
Phylogram based on a combined ITS, LSU SSU, RPB2 and TEF1-α sequence data of selected members of four orders of the Savoryellomycetidae. Bootstrap support values for maximum likelihood (ML) greater than 70% and Bayesian posterior probabilities (PP) greater than 0.95 are given as ML/PP above the nodes. Newly obtained sequences are indicated in red and ex-type strains are in bold.

In the phylogenetic analysis, *Dematipyriformamuriformis* (MFLU 21–0146) clustered with the ex-type strain of *D.aquilaria* (CGMCC 3.17268) with low support (Fig. 1). The new isolate of *Rhexoacrodictysfimicola* (MFLUCC 18–0340) clustered with three strains of *R.fimicola* (HMAS 42882, HMAS 43690 and HMAS 47737) with 100% ML/1.00 PP support (Fig. 1). *Pleurotheciumaquaticum* (KUNCC 21–0477) clustered with the ex-type strain of *P.aquaticum* (MFLUCC 17–1331) with 100% ML/1.00 PP support (Fig. 1).

### ﻿Taxonomy

#### ﻿ Dematipyriforma muriformis

Taxon classificationFungiPleurothecialesPleurotheciaceae

D.F. Bao, K.D. Hyde & Z.L. Luo sp. nov.

9771DF07-5B27-5031-9B8E-090279283AA0

http://www.indexfungorum.org/names/NamesRecord.asp?RecordID=553383

https://www.facesoffungi.org/?s=FoF10414

Fig. 2

##### Etymology.

Referring to the muriform conidia of this species.

##### Holotype.

MFLU 21–0146.

##### Description.

*Saprobic* on submerged decaying wood. Sexual morph: Undetermined. Asexual morph: *Colonies* on substratum superficial, scattered, black, shining, granulate. *Mycelium* immersed, composed of hyaline, branched, septate, smooth, hyphae. *Conidiomata* sporodochial, subhyaline. *Conidiophores* 10–26.5 × 2–3 μm (*x*‒ = 18.2 × 2.3 μm, n = 20), micronematous to semi-macronematous, mononematous, fasciculate, simple or branched, hyaline, cylindrical, smooth. *Conidiogenous cells* monoblastic, integrated, terminal, determinate, hyaline, smooth. *Conidia* 23–26 × 15.5–18 μm (*x*‒ = 24.6 × 16.7 μm, n = 30), acrogenous, solitary, smooth, thick-walled, ellipsoidal to obovoid, muriform, rounded at apex, pointed at base, with 3–5 transverse septa, 1-longitudinal septum in all cells and rarely in end cells, slightly constricted at septa, subhyaline to pale olivaceous when young, olive to dark brown at maturity.

##### Material examined.

Thailand, Bangkok Province, Bang Kapi District, on decaying wood submerged in a freshwater stream, 3 October 2017, Z.L. Luo, Bsite 4–3–1 (MFLU 21–0146, holotype; KUN-HKAS 122858, isotype).

##### Notes.

In the phylogenetic analysis, *Dematipyriformamuriformis* clustered with the ex-type strain of *D.aquilaria* (CGMCC 3.17268) within Pleurotheciaceae with low support (Fig. 1). The ITS blast result in NCBI GenBank showed that *D.muriformis* (MFLU 21–0146) is 92.36% and 91.92% similar to *D.nigrospora* (MFLUCC 21-0097) and *D.aquilaria* (CGMCC 3.17268) respectively.

*Dematipyriformamuriformis* resembles *D.aquilaria* in having micronematous, mononematous, smooth septate conidiophores, monoblastic, integrated, terminal, determinate conidiogenous cells and solitary, muriform conidia. However, *D.muriformis* differs from *D.aquilaria* in having hyaline conidiophores and slightly smaller conidia (23–26 × 15.5–18 vs. 25–37.5 × 15–22.5 μm). In addition, conidia of *D.muriformis* are subhyaline to pale olivaceous when young, olive to dark brown at maturity, with 3–5 transverse septa, 1-longitudinal septum in all cells and rarely in end cells. Whereas, *D.aquilaria* has pale grey olivaceous to pale brown conidia with 4–5 transverse septa and 0–2 longitudinal septa (Sun et al. 2017).

*Dematipyriformamuriformis* shares some similar characteristics with *Neomonodictys* taxa in Pleurotheciaceae, such as monoblastic, integrated, terminal, determinate conidiogenous cells and muriform conidia. *Neomonodictys*, however, lacks sporodochial conidiomata and conidia of *Neomonodictys* are subglobose to globose, while, *Dematipyriformamuriformis* has ellipsoidal to obovoid conidia (Hyde et al. 2020b).

**Figure 2.**
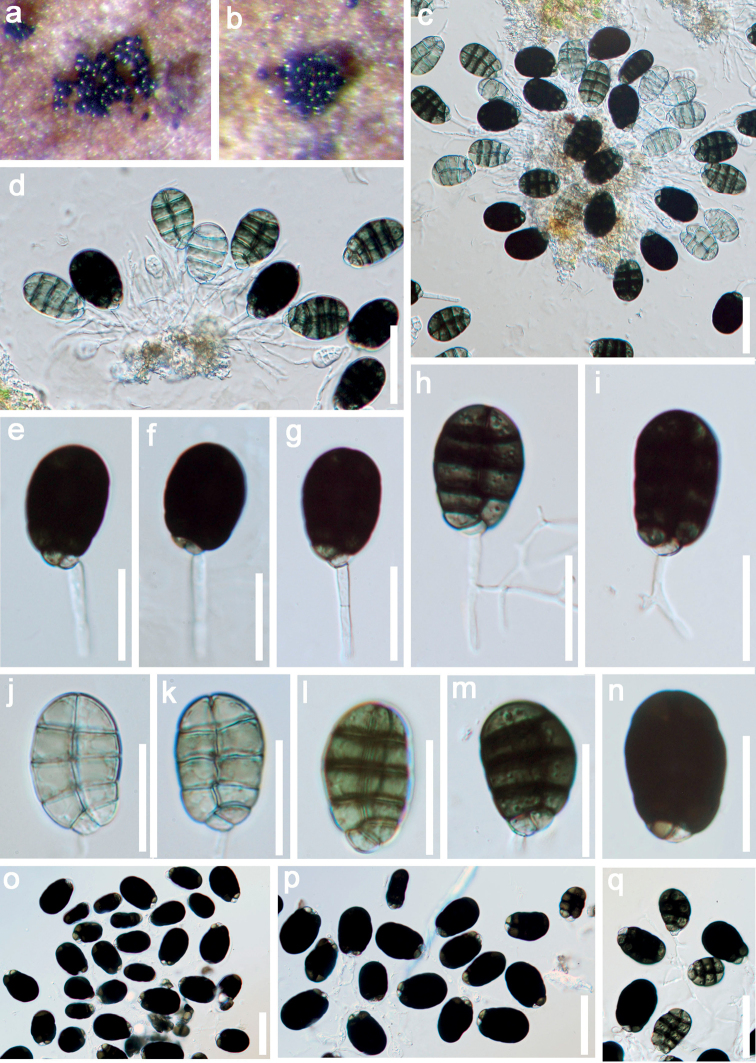
*Dematipyriformamuriformis* (MFLU 21–0146, holotype) **a, b** colonies on wood **c–d** conidiomata **e–i** conidiophore with conidia **j–q** conidia. Scale bars: 30 μm (**c–d, o–q**); 20 μm (**e–n**).

#### ﻿ Dematipyriforma nigrospora

Taxon classificationFungiPleurothecialesPleurotheciaceae

(Boonmee, D.F. Bao & K.D. Hyde) D.F. Bao, K.D. Hyde & Z.L. Luo comb. nov.

A6148170-3B36-5B96-9677-CAD862AE897E

http://www.indexfungorum.org/names/NamesRecord.asp?RecordID=553384

≡ Rhexoacrodictysnigrospora Boonmee, D.F. Bao & K.D. Hyde, in Boonmee et al., Fungal Diversity 111: 200 (2021).

##### Holotype.

Thailand, Phetchabun Province, on decaying bark, 25 July 2019, S. Boonmee, LSP03 (MFLU 21–0073).

##### Descriptions and illustrations.

See Boonmee et al. (2021).

##### Notes.

*Rhexoacrodictysnigrospora* was introduced by Boonmee et al. (2021) based on morphological characters and phylogenetic analysis. In our phylogenetic analysis, *R.nigrospora* clustered with two *Dematipyriforma* species (*D.aquilaria and D.muriformis*) in a distinct clade within Pleurotheciaceae (Fig. 1). Therefore, we transfer *Rhexoacrodictysnigrospora* to *Dematipyriforma*, as *Dematipyriformanigrospora* comb. nov.

*Dematipyriformanigrospora* resembles *D.muriformis* in having micronematous or semi-macronematous, mononematous conidiophores and monoblastic, polyblastic, integrated, terminal conidiogenous cells. However, *D.nigrospora* differs from *D.muriformis* in having brown to dark brown conidiophores and globose to subglobose, dark brown to black conidia (Boonmee et al. 2021). Conidiophores of *D.muriformis* are hyaline and conidia are ellipsoidal to obovoid, muriform, and subhyaline to pale olivaceous when young, olive to dark brown at maturity.

#### ﻿ Rhexoacrodictys fimicola

Taxon classificationFungiPleurothecialesPleurotheciaceae

(M.B. Ellis & Gunnell) W.A. Baker & Morgan-Jones, in Baker, Partridge & Morgan-Jones, Mycotaxon 82: 103 (2002)

3D33BD6A-675E-542C-8B19-614ECB4162C6

Fig. 3

##### Holotype.

Maya, Perak, on elephant dung, September 1958, A.H.S, Onions, IMI 76413.

##### Description.

*Saprobic* on submerged decaying wood. Sexual morph: Undetermined. Asexual morph: *Colonies* on the substratum superficial, effuse, hairy or velvety, black. *Mycelium* mostly immersed, composed of branched, septate, smooth, pale brown hyphae. *Conidiophores* (17.5–)20–44.5 (–65.5) × 2.5–4.0 μm (*x*‒ = 32.2 × 3.4 μm, n = 20), macronematous, mononematous, erect, straight or slightly flexuous, thick-walled, smooth, orange-brown or brown, 3–7-septate. *Conidiogenous cells* monoblastic, integrated, terminal. *Conidia* 16.5–24 × 11–15 μm (*x*‒ = 20.3 × 13 μm, n = 30), solitary, dry, acrogenous, broadly oval to subglobose, muriform, transversely and longitudinally septate, with transverse septa typically spanning the whole conidial width, with longitudinal septa typically incomplete, short; dark-blackish brown to black, smooth, narrowly truncate at the base.

**Figure 3.**
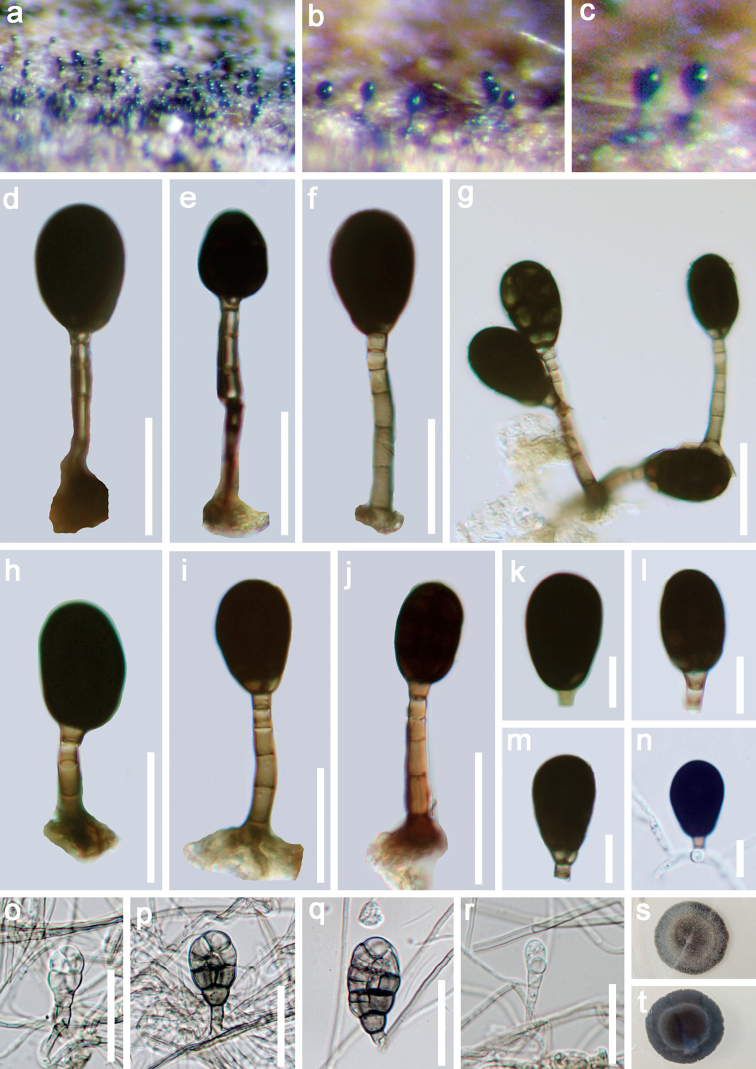
*Rhexoacrodictysfimicola* (MFLU 21–0147, new record) **a–c** colonies on wood **d–j** conidiophores with conidia **k–m** conidi **n** germinating conidium **o–r** re-produced asexual morph of *Rhexoacrodictysfimicola***s–t** culture on PDA from surface and reverse. Scale bars: 20 μm (**d–j, o–r**); 10 μm (**k–n**).

##### Cultural characteristics.

*Conidia* germinating on PDA within 24 h. Germ tubes produced from the basal cell. *Colonies* on PDA reaching 3 cm diameter in 30 days at 20–25 °C, pale brown, with dense, tight mycelia on the surface, sparse at the margin, reverse dark brown, with smooth margin. *Conidiophores* reduced to conidiogenous cells. *Conidiogenous cells* holoblastic, monoblastic, integrated, hyaline to pale brown, smooth. *Conidia* broad oval to subglobose, muriform, strongly constricted at all the septa, hyaline when young, brown to grayish-brown when aged, smooth-walled.

##### Material examined.

Thailand, Bangkok Province, Bang Kapi District, on decaying wood submerged in a freshwater stream, 3 October 2017, Z.L. Luo, Bsite 4–3–2 (MFLU 21–0147 = KUN-HKAS 122859), living culture, MFLUCC 18–0340.

##### Notes.

In the phylogenetic analysis, our new isolate MFLUCC 18–0340 clustered with three strains of *Rhexoacrodictysfimicola* (HMAS 42882, HMAS 43690 and HMAS 47737) with strong support (100% ML/ 1.00 PP). The nucleotide BLASTn search of ITS showed that our new strain (MFLUCC 18–0340) has 99.7%, 99.3% and 99.1% similarities with strain HMAS 43690, HMAS 47737 and HMAS 42882 of *Rhexoacrodictysfimicola*, respectively. Morphologically, our new collection is similar to *R.fimicola* in having macronematous, mononematous, indeterminate conidiophores, integrated, terminal, monoblastic, pale brown conidiogenous cells and broadly oval to subglobose, transversely and longitudinally septate, smooth, brown to black conidia, with the size of conidia and conidiophores are overlapping (Ellis 1961; Baker et al. 2002). Based on both phylogeny and morphology, we identified our species as *R.fimicola*.

*Rhexoacrodictysfimicola* was originally introduced by Ellis (1961) as *Acrodictysfimicola*. Baker et al. (2002) transferred *A.fimicola* to *Rhexoacrodictys* based on morphological characteristics. *Rhexoacrodictysfimicola* has been reported on *Bambusavulgaris* and elephant dung from Africa and Malaysia respectively. Our collection, on the other hand, was collected from freshwater habitats and represents the first time it was reported from Thailand.

#### ﻿ Pleurothecium aquaticum

Taxon classificationFungiPleurothecialesPleurotheciaceae

Z.L. Luo, H.Y. Su & K.D. Hyde, in Luo, Hyde, Bhat, Jeewon, Maharachchikumbura, Bao, Li, Su, Yang & Su, Mycol. Prog. 17(5): 526 (2018)

7644B4BA-2F53-5575-A238-F9CE05C64759

Fig. 4

##### Description.

*Saprobic* on submerged decaying wood. Sexual morph: Undetermined. Asexual morph: *colonies* on substratum, effuse, shining, dark brown to black. *Mycelium* partly immersed, composed of septate, branched, smooth, dark brown hyphae. *Conidiophores* 84–110 × 3–4 μm (*x*‒ = 97 × 3.4 μm, n = 10), macronematous, mononematous, erect, simple, unbranched, straight or slightly flexuous, 5–8-septate, dark brown, pale towards apex, smooth. *Conidiogenous cells* integrated, polyblastic, terminal, hyaline, denticulate, smooth. *Conidia* 18–22 × 4–5 μm (*x*‒ = 20 × 4.5 μm, SD = 4 n = 30), acrogenous, solitary, clavate, mostly curved, rounded at apex, tapering at base, hyaline, 3-septate, with guttulate cells, smooth.

##### Cultural characteristics.

*Conidia* germinating on PDA within 24 h. Germ tubes produced from the basal and apical cells. *Colonies* on PDA reaching 2.3 cm diameter in 30 days at 20–25 °C, with dense mycelia, dry, rigid, rugose, dark brown, reverse dark brown.

##### Material examined.

Thailand, Prachuap Khan, on submerged decaying wood, 15 August 2017, V. Kumar, site1–24–2 (MFLU 21–0148 = KUN-HKAS 122857), living culture, KUNCC 21–0477.

##### Notes.

In the phylogenetic analysis, our new collection KUNCC 21–0477 clustered with the ex-type strain of *Pleurotheciumaquaticum* (MFLUCC 17–1331) with high (100% ML/1.00 PP). In addition, the ITS and LSU BLASTn search on NCBI GenBank showed that our new strain is 99.88% and 97.45% similarities to the ex-type of *P.aquaticum* (MFLUCC 17–1331). The new collection is morphologically similar to *P.aquaticum* in having macronematous, mononematous, septate, brown, pale brown towards the apex conidiophores, integrated, terminal, polyblastic, denticulate conidiogenous cells and hyaline, cylindrical or clavate, rounded at the apex, obtuse and tapering towards base, 3-septate conidia. We therefore identified our new collection as *P.aquaticum*. *Pleurotheciumaquaticum* was introduced by Luo et al. (2018a) collected from freshwater habitats in China. Our new collection, on the other hand, was collected from Thailand and is a new record for Thailand.

**Figure 4.**
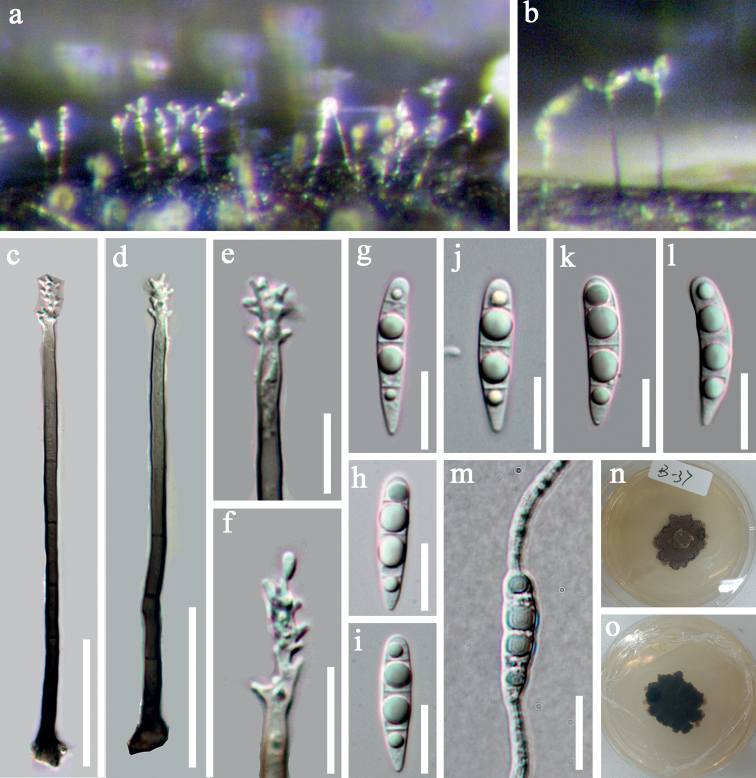
*Pleurotheciumaquaticum* (MFLU 21–0148, new record) **a, b** colonies on wood **c, d** conidiophores **e, f** conidiogenous cells **g–i** conidia **m** germinating conidium **n, o** culture on PDA from surface and reverse. Scale bars: 30 μm (**c, d**); 10 μm (**e–m**).

## ﻿Discussion

Pleurotheciaceae is a diverse family. The sexual morphs of Pleurotheciaceae are quite similar and difficult to distinguish without molecular data (Réblová et al. 2016; Hyde et al. 2020a). However, the asexual morphs in the family are morphologically diverse. Most genera have mononematous, macrounematous conidiophores (*Anapleurothecium*, *Pleurothecium*, *Pleurotheciella* and *Rhexoacrodictys*) (Réblová et al. 2016; Luo et al. 2018a, 2019; Hyde et al. 2020a), whereas some genera have synnematous conidiophores (*Phaeoisaria* and *Phragmocephala*) (Höhnel 1919; Mason and Hughes 1951; Seifert et al. 2011; Wijayawardene et al. 2012; Su et al. 2015; Réblová et al. 2016; Luo et al. 2018a), and others with micronematous or reduced conidiophores (*Neomonodictys* and *Sterigmatobotrys*). (Hyde et al. 2020b). Conidiogenous cells of *Anapleurothecium*, *Pleurothecium*, *Phaeoisaria* and *Pleurotheciella* are polyblastic and denticulate (Réblová et al. 2012, 2016; Monteiro et al. 2016; Luo et al. 2018a). *Phragmocephala* and *Monotosporella* have monoblastic conidiogenous cells (Mason and Hughes 1951; Hyde and Yanna 2002; Wijayawardene et al. 2012; Su et al. 2015). Conidia of Pleurotheciaceae are diverse in their shape, color and septation. Conidia of *Sterigmatobotrys* are fusiform and in persistent chains (Heuchert et al. 2018); *Helicoascotaiwania* has helicosporous conidia (Dayarathne et al. 2019); *Anapleurothecium*, *Melanotrigonum*, *Pleurothecium*, *Phaeoisaria* and *Pleurotheciella* have clavate, ellipsoidal, obovoidal, fusiform-cylindrical, hyaline or brown, aseptate or transversely septate conidia (Réblová et al. 2012, 2016; Monteiro et al. 2016; Hernandez-Restrepo et al. 2017; Luo et al. 2018a); *Monotosporella*, *Neomonodictys* and *Phragmocephala*have ellipsoidal or subglobose to globose conidia (Mason and Hughes 1951; Hyde and Yanna 2002;Wijayawardene et al. 2012; Su et al. 2015; Hyde et al. 2020b). However, conidia of *Neomonodictys* are muriform (Hyde et al. 2020b), whereas, *Phragmocephala* and *Monotosporella* have transversely septate conidia.

In this study, we introduced a new asexual species, *Dematipyriformamuriformis* based on both morphology and phylogeny. *Dematipyriforma* was introduced by Sun et al. (2017) with a single species *D.aquilaria* which was reported as an endophyte from *Aquilariacrassna* in China. While our new species is a saprobe isolated on submerged wood from freshwater habitats in Thailand. In addition, *Rhexoacrodictysnigrospora* is transferred to *Dematipyriforma* in this study. Currently, three species are accepted in the genus. Morphologically, the muriform conidia of *Dematipyriforma* are similar to *Neomonodictys*, *Saprodesmium* and *Coleodictyospora*. However, *Dematipyriforma* can be distinguished from *Neomonodictys* by the shape of conidia (ellipsoidal to obovoid vs. subglobose to globose) and conidiophores (semi-micronematous to macronematous vs. micronematous or lacking conidiophores, Hyde et al. 2020b). *Dematipyriforma* differs from *Coleodictyospora* in the conidia lacking a semi-gelatinous sheath (Dong et al. 2021). *Dematipyriforma* is distinct from *Saprodesmium* by the micronematous to semi-macronematous, simple or branched, hyaline, cylindrical, conidiophores, whereas, conidiophores of *Saprodesmium* are micronematous, unbranched, consisted of 1–4 subglobose smooth, hyaline cells (Dong et al. 2021).

*Rhexoacrodictys* comprises six species of which four species (*R.erecta*, *R.fimicola*, *R.martini* and *R.queenslandica*) have sequence data available in the GenBank. Among them, *R.martini* and *R.queenslandica* were transferred to *Distoseptispora* and *Junewangia* based on phylogenetic analysis (Xia et al. 2017). However, sequence data of *R.martini* are doubted by several studies (Sun et al. 2020; Shen et al. 2021), as its morphology does not fit with the characters of *Distoseptispora*. *Rhexoacrodictyserecta* and *R.fimicola* clustered within Pleurotheciaceae (Luo et al. 2019; Dong et al. 2021). The placement of *Rhexoacrodictys* was questionable since it was established. Baker et al. (2002) established the genus; however, they did not mention the placement of the genus. Xia et al. (2017) firstly provided sequence data for *Rhexoacrodictyserecta* (Type species of *Rhexoacrodictys*) and *R.fimicola* based on their fresh collections, their phylogenetic analysis showed that *R.*erecta and *R.fimicola* clustered within Savoryellaceae. However, they did not include the related orders (Conioscyphales, Fuscosporellales and Pleurotheciales) in Savoryellomycetidae. Luo et al. (2019) found that *R.*erecta and *R.fimicola* grouped in Pleurotheciaceae. Recently, Dong et al. (2021) obtained the same result as Luo et al. (2019). However, Boonmee et al. (2021) and Wijayawardene et al. (2022) placed *Rhexoacrodictys* in Savoryellaceae (Savoryellales). Our result is consistent with Luo et al. (2019) and Dong et al. (2021), the two species clustered within Pleurotheciaceae (Fig. 1). On the other hand, the morphology of *Rhexoacrodictys* is similar to *Dematipyriforma*, *Neomonodictys* and *Saprodesmium*, in having muriform conidia, micronematous conidiophores and holoblastic, monoblastic conidiogenous cells. Therefore, we formally accepted *Rhexoacrodictys* in Pleurotheciaceae (Pleurotheciales) based on morphological characters and phylogenetic analysis.

In our phylogenetic analysis, *Rhexoacrodictyserecta* and *R.fimicola* clustered with *Monotosporellasetosa* which is the type species of *Monotosporella*. Morphologically, *R.erecta* and *R.fimicola* fit well within the genus concept of *Monotosporella* in having macronematous, mononematous, brown, septate conidiophores, monoblastic, percurrent conidiogenous cells and acrogenous, brown septate conidia (Hughes 1958; Baker et al. 2002; Hyde and Yanna 2002). However, the strain of *Monotosporellasetosa* (HKUCC 3713) lacks a morphological description. Therefore, further study is necessary to clarify the relationship between *Rhexoacrodictys* and *Monotosporella*, whether they should be combined into one genus or not. In addition, our phylogenetic analysis showed that three strains of *R.erecta* clustered with *Monotosporellasetosa*. However, *M.erecta* differs from *M.setosa* in having transverse and longitudinal septation, while, conidia of *M.setosa* only have transverse septa. Only LSU sequence data is available for *M.setosa*, which is not significant to distinguish in the phylogenetic tree, but morphologically they are quite distinct. Hence, we maintain them as two distinct species, however, further morphological and phylogenetic analysis is required to clarify the relationship between these two species.

In our phylogenetic analysis, *Pleurotheciumobovoideum* was placed distant from *Pleurothecium* and close to *Neomonodictysmuriformis* and *Coleodictyosporamuriformis* which is consistent with recent studies (Luo et al. 2018a, 2019; Hyde et al. 2020b). *Pleurotheciumobovoideum* was introduced by Arzanlou et al. (2007) based on morphological characters. However, their analysis showed that *P.obovoideum* clustered with *Ascotaiwaniahughesii* and with more sequence data now available for *Pleurothecium* species, *P.obovoideum* is shown phylogenetically distinct from *Pleurothecium*. Morphologically, *P.obovoideum* is similar to *Pleurothecium* in having distinct brown conidiophores, polyblastic, denticulate conidiogenous cells and pale brown, ellipsoidal to obovate conidia. However, conidia of *P.obovoideum* are aseptate and solitary or in short chains whereas the conidia of *Pleurothecium* are solitary and unicellular or septate. Thus, the placement of *P.obovoideum* needs revision in the future with more evidence.

## Supplementary Material

XML Treatment for Dematipyriforma muriformis

XML Treatment for Dematipyriforma nigrospora

XML Treatment for Rhexoacrodictys fimicola

XML Treatment for Pleurothecium aquaticum
